# Comparing forces on the fetal neck in breech delivery in lithotomy versus all-fours position: a simulation model

**DOI:** 10.1007/s00404-022-06671-5

**Published:** 2022-07-20

**Authors:** Delnaz Fard, Chiara S. Borchers, Jill-Caren Philippeit, Anja V. Philippeit, Laura R. Kaukemüller, Lara R. Higgins-wood, Spyridon Papageorgiou, Peter Hillemanns, Constantin S. von Kaisenberg, Rüdiger Klapdor

**Affiliations:** Department of Obstetrics, Gynecology and Reproductive Medicine, Hanover Medical School, Carl-Neuberg-Str. 1, 30625 Hanover, Germany

**Keywords:** All-fours position, Breech delivery, Brachial plexus, Lithotomy position, Simulator, Spontaneous vaginal breech delivery

## Abstract

**Purpose:**

To measure forces applied to the fetal neck, in a simulation model for breech delivery, in both lithotomy versus all-fours position.

**Methods:**

We used a Laerdal SimMom simulator and a Birthing Baby together with PROMPT Flex Software. The descent of the fetus was accomplished using the Automatic Delivery Module 2. The baby was always in breech position; the SimMom in either all-fours or lithotomy positions. Sensors were located inside the fetal neck region to simulate forces applied to the plexus.

**Results:**

The lowest force on the fetal neck region was recorded for the delivery in all-fours position without further maneuvers (mean force 58.70 Newton, standard deviation 2.54 N). As weight was added to the baby, the force increased (i.e. + 500 g, mean force 71.8 N, SD 3.08 N, *p* < 0.001). Delivery in lithotomy position resulted in a mean force of 81.56 N (SD 19.55 N). The force significantly increased in case of delivery of the head without assistance from contractions (mean force 127.93 N, SD 23.10 N). In all-fours position, the delivery of the fetal head from pelvic floor level without contractions (Frank’s Nudge maneuver) resulted in a mean force of 118.45 N (SD 15.48 N, *p* = 0.02). Maneuvers for shoulder dystocia (the inverted type that can occur during breech delivery) led to significantly higher mean forces independent from birthing positions.

**Conclusion:**

Breech delivery in all-fours position was associated with the lowest force acting on the fetal neck in our simulation model.

**Supplementary Information:**

The online version contains supplementary material available at 10.1007/s00404-022-06671-5.

## Introduction

What does this study add to the clinical work Force applied to the fetal neck during breech delivery in lithotomy and all-fours position could be measured for the first time in a simulation model. In uncomplicated breech deliveries, the use of the all-fours position was associated with a lower level of applied forces compared to delivery in lithotomy and was not dependent on the physician’s experience."

Spontaneous vaginal breech delivery is still a controversial issue although the number of vaginal deliveries has increased in the last years. The Term Breech Trial reported a significant reduction of perinatal mortality and morbidity for fetuses in breech presentation delivered by planned cesarean section compared to planned vaginal delivery (RR 0.33 [95% CI 0.19–0.56]; *p* < 0.0001) [1]. The Term Breech Trial was widely criticized for methodical aspects of the study [2, 3]. In contrast, several studies and reviews have shown identical outcomes for planned cesarean section versus vaginal breech delivery [4–7]. The current mainstream opinion is that vaginal breech and cesarean section appear to have similar outcomes, as long as inclusion and exclusion criteria are applied and strict indications and restrictions are adhered to [5, 8, 9].

Apart from fetal hypoxia due to the delayed delivery of the fetal head, breech delivery can be associated with perinatal or permanent brachial plexus injury [4]. Several studies are indicating that breech delivery is one of the most important risk factors for plexus palsy [10, 11]. There are some preliminary studies which show that the all-fours/upright position is a favorable setting and a realistic alternative to the entrenched lithotomy position [12–14]. It has been assumed that maternal movements and the influence of gravity seem to facilitate the fetal descent and appears to be associated with a reduction of the need for intervention and therefore reduces the force applied to the fetal neck [12].

There are no data from simulation models directly measuring forces applied to the fetal neck or looking at the impact of the birth position, the fetal weight and the maneuvers applied. Therefore, scientific data are needed.

The objective of this study is to measure forces and compare the different birth positions and maneuvers objectively for the forces applied to the fetal neck. The model used can serve as a basis for future studies to better understand how to optimize and train vaginal breech delivery and to give feedback for the forces applied.

## Materials and methods

In this prospective study, we investigated the force on the fetal neck region applied during delivery in fetal breech presentation. The study was performed using a SimMom (Laerdal, Puchheim, Germany), which is a full body high fidelity obstetrical manikin allowing to simulate descent and birth of the baby in the various maternal positions in a standardized way. The Automatic Delivery Module 2 (Laerdal, Puchheim, Germany) enabled fully automatic and standardized birth processes and simulation of contractions. The simulation baby, Birthing Baby (Laerdal, Puchheim, Germany), allowed a measurement and recording of forces applied to the baby’s neck region during interventions. The Birthing Baby was equipped with an internal electronic strain gauge as part of the standard configuration by Laerdal. This worked in conjunction with the force monitoring software (PROMPT Flex, v0.179.0). The location of the sensors was inside the neck thus representing brachial plexus.

The simulation was performed by five obstetricians:

One senior physician with qualification in special obstetrics, one consultant with experience in vaginal breech deliveries, two other residents with at least 2 years of training in deliveries and obstetrics and one junior resident with no training.

**Fig. 1:**
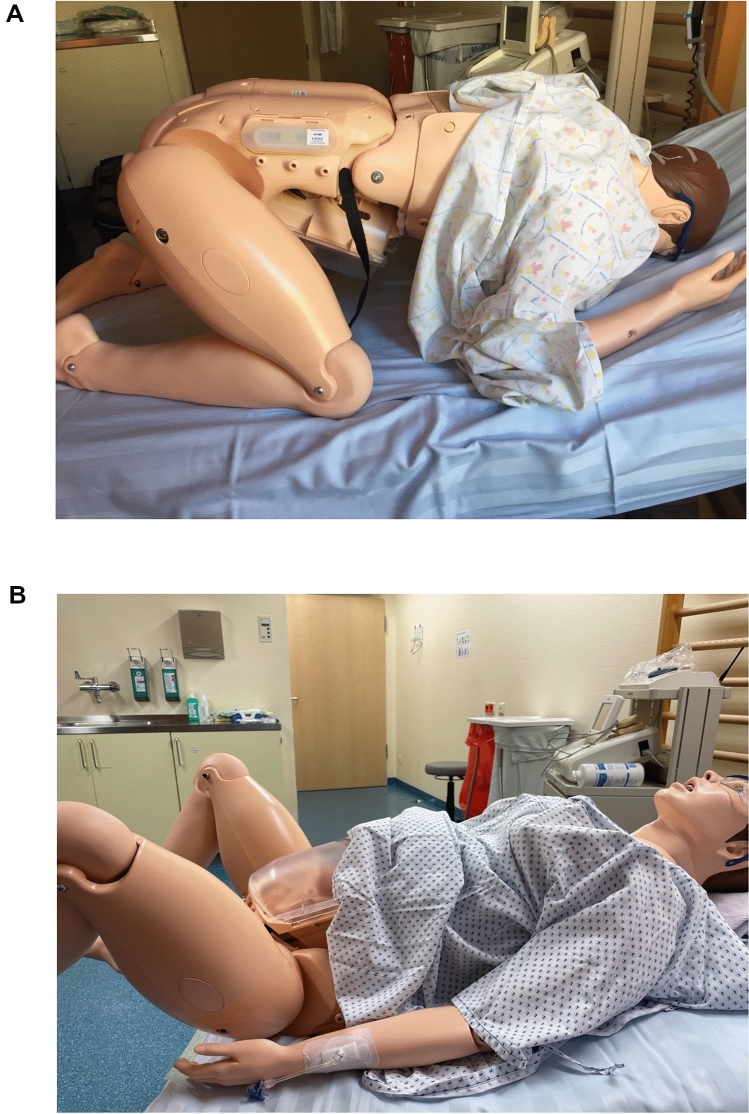
Experimental setup with the full body obstetrical simulator in all-fours (**A**) and in lithotomy position (**B**)

In the preliminary phase, all physicians received instructions regarding the maneuvers and childbirth positions. The physicians were allowed to practice the maneuvers in a test run without getting feedback on the applied forces. All physicians went through scenarios of uncomplicated (I) and complicated (II) deliveries (*Supplement 1*).

(I) For uncomplicated deliveries, we analysed the delivery in all-four position with and without addition of 500 g, 1000 g and 1500 g weight to the fetal body (model fetal weight: 2080 g) to evaluate the effect of the fetal body weight on forces during all-fours position (Fig. 1A). Additionally, we evaluated the Bracht maneuver for delivery in lithotomy position, which mirrors the delivery in all-fours position and is the routine procedure in Germany (Fig. 1B). Both above-mentioned maneuvers were performed with support of simulated contractions.
(II) For complicated deliveries, we evaluated the force in lithotomy position and all-fours position (Frank’s Nudge maneuver) in emergency situations when the head had to be delivered without the support of simulated contractions. In another situation, we evaluated maneuvers in the event of shoulder dystocia (the inverted type that can occur during breech delivery) in all-fours (rotation maneuver) and lithotomy position (Bickenbach maneuver) (*Supplement 1*).

All scenarios were repeated 10 times by each physician. The applied forces to the fetal neck region were recorded in real time and were stored digitally using the PROMPT Flex software. Further analyses were performed using Microsoft Excel Version 14.4.7 (Redmont, USA). For statistical evaluation, all data were stored in a database. Mean forces and standard deviations of the applied force were calculated. The paired *t* test was used for calculation of *p* values of continuous data. A *p* value below 0.05 was considered to be significant. The study was approved by the Local Ethical Committee of the Hanover Medical School (Nr. 9600_BO_K_2021), 5th February 2021. The five obstetricians gave informed consent to participate in the study.

## Results

We grouped the results in uncomplicated and complicated breech deliveries.

At first, we evaluated and compared the forces affecting the fetal neck region during uncomplicated breech deliveries in all-fours and lithotomy positions (Fig. 1A, B).

**Fig. 2:**
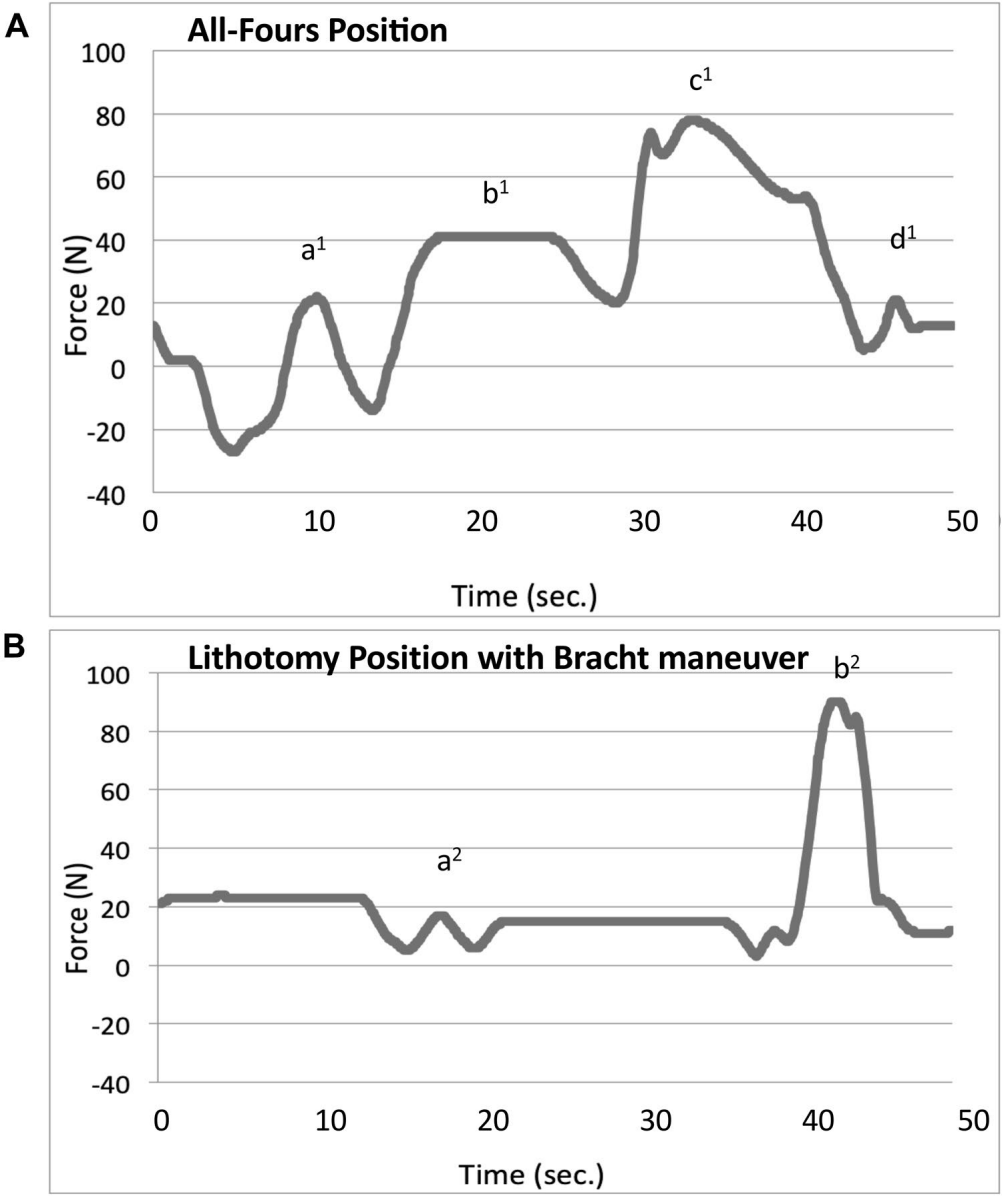
Forces measured in the fetal neck over time in uncomplicated deliveries. **A** Exemplary run-in all-fours position without application of external force and increased fetal bodyweight by application of a 500 g additional weight around the fetal belly. a^1^ Navel visible; b^1^ scapule visible; c^1^ spontaneous birth of arms; d^1^ spontaneous birth fetal head; [N] Newton. **B** Exemplary curve of a setup in lithotomy position while using the Bracht maneuver, which is the routine procedure in Germany. a^2^ Scapule visible; b^2^ spontaneous birth, fetal head; [N] Newton

**Fig. 3:**
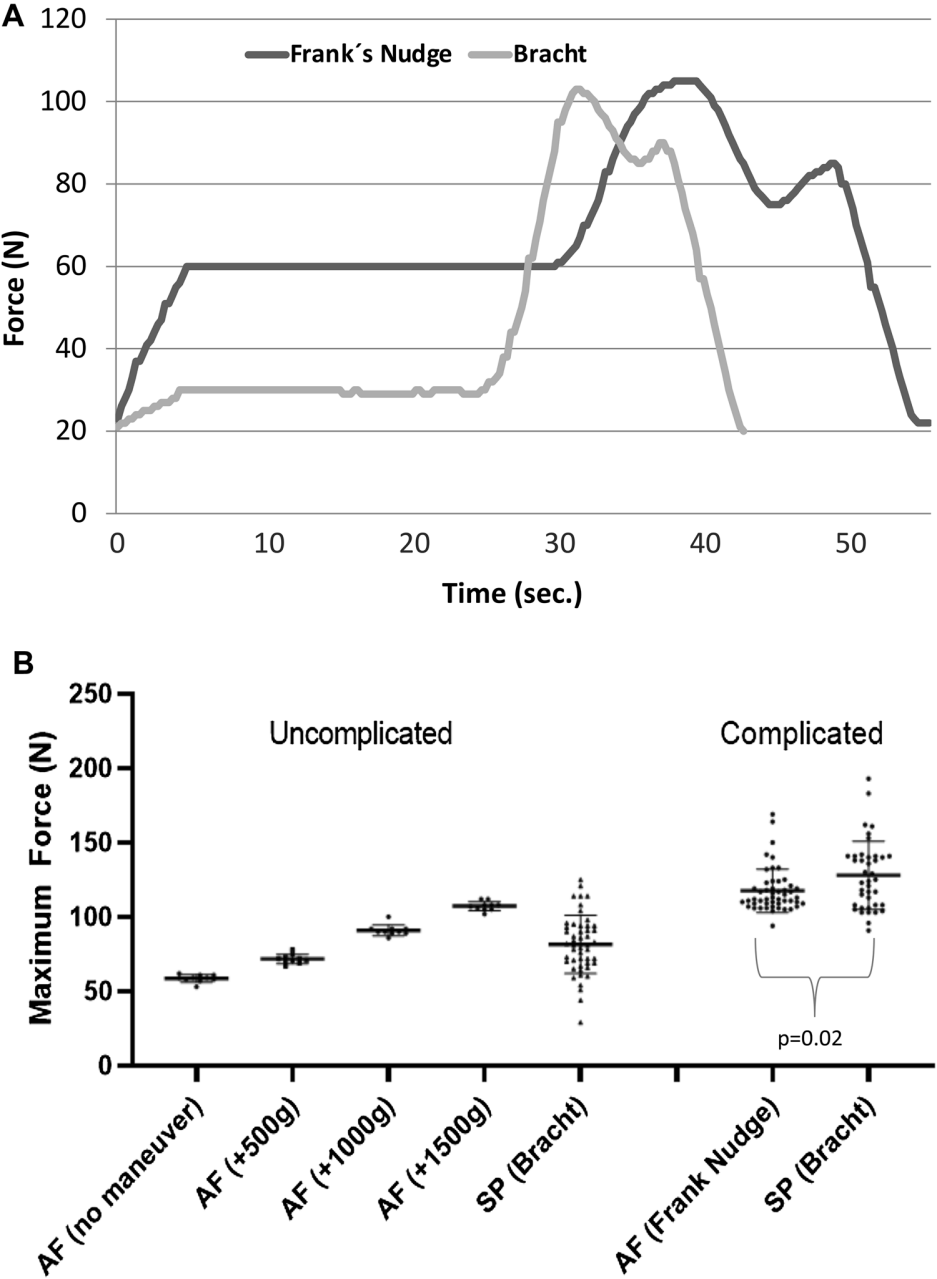
Forces measured in the fetal neck over time and in comparison, in uncomplicated and complicated deliveries. **A** Exemplary run: comparison of the forces affecting the fetal neck region with either the Frank’s Nudge [all-fours] or Bracht maneuvers [lithotomy position] from pelvic floor level without contractions. **B** Plots of maximum force levels in uncomplicated deliveries in all-fours position and in lithotomy position using the Bracht maneuver and complicated deliveries without contractions using Frank Nudge [all-fours] or Bracht [lithotomy position] maneuver from pelvic floor level. The performance of each maneuver was operator depended [Kruskal–Wallis test *p* < 0.01]. *AF* all-fours, *SP* lithotomy position, *[N]* Newton

Uncomplicated delivery simulation in all-fours position was performed without application of external force or maneuvers (Fig. 2A). The lowest force affecting the fetal neck region was recorded for the spontaneous delivery in all-fours position without the need of any further maneuver (mean force 58.70 N, SD 2.54 N) (Fig. 3B on the left). We also evaluated the effect of additional bodyweight. Added weight of 500 g, 1000 g and 1500 g proportionally increased the measured force to 71.8, 91.0 and 107.3 N, respectively (Fig. 3B).

The Bracht maneuver, which mirrors the spontaneous delivery in all-fours position, was used in lithotomy position, since it is the standard procedure applied in uncomplicated breech deliveries in Germany (Fig. 2B). We recorded a mean force of 81.56 N (SD 19.55 N) in uncomplicated deliveries with contractions.

To evaluate the maximum applied force in emergency situations, a simulation was created in which the contractions stopped after delivery of the shoulders. For both birthing positions, as expected, forces on the fetal neck significantly increased when maneuvers were applied. We detected a higher force in lithotomy position compared to the all-fours position (mean force 127.93 N vs 118.45 N, p = 0.02) (Fig. 3A and B).

Using the Veit–Smellie–Mauriceau maneuver for the head delivery in lithotomy position led to a mean value of 74.6 N (SD 9.16). This was significantly lower compared to the Bracht maneuver in lithotomy position, as well for uncomplicated deliveries (*p* = 0.003) as for complicated deliveries (*p* < 0.001) and the Frank’s Nudge maneuver in all-fours position (*p* < 0.001 for complicated deliveries).

**Fig. 4:**
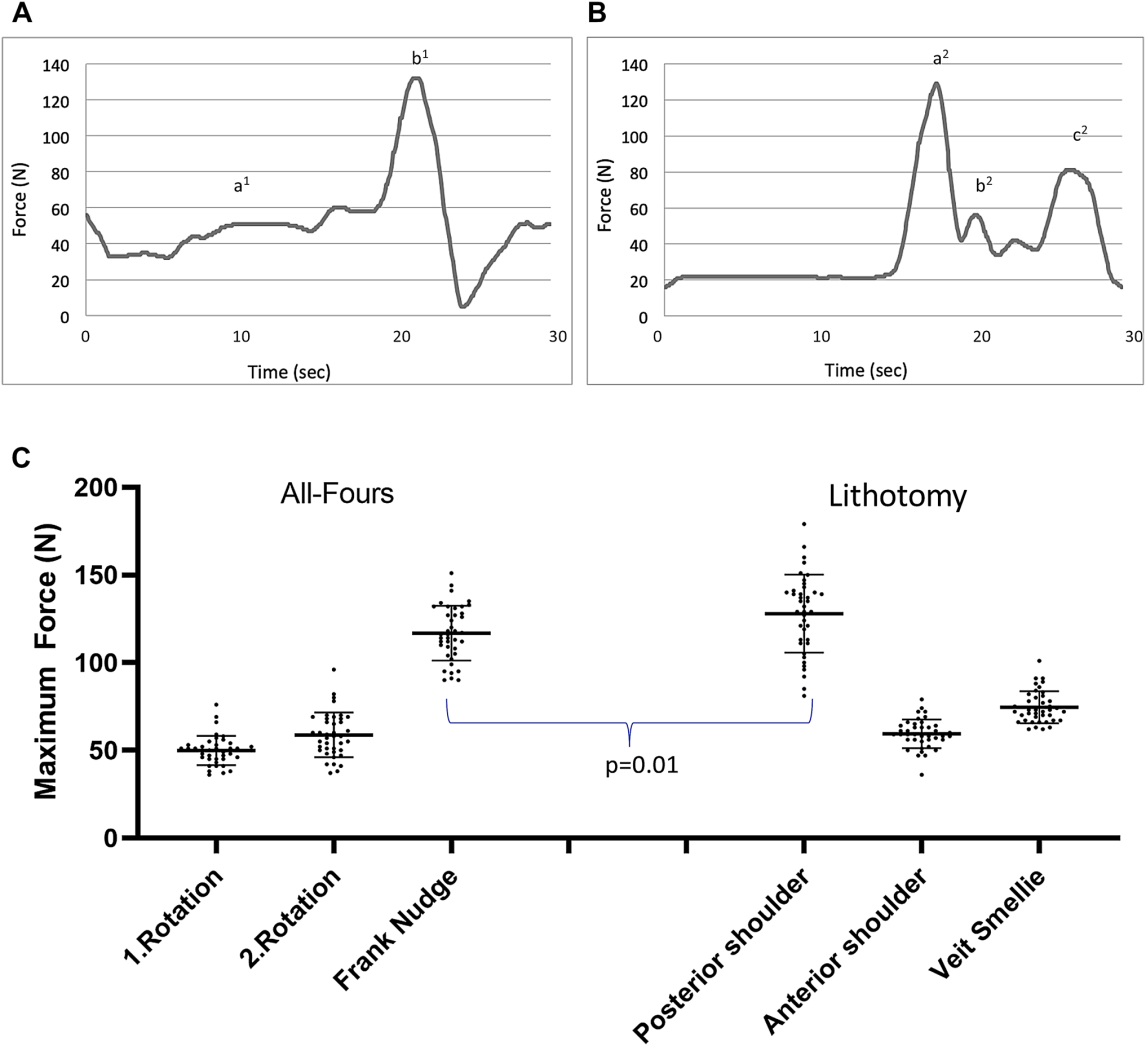
Forces measured in the fetal neck over time for shoulder dystocia in all-fours position. And lithotomy position. **A**, **B** Exemplary curves of runs with shoulder dystocia in all-fours position (**A**) and Bickenbach maneuver with Veit–Smellie–Mauriceau maneuver in lithotomy position (**B**). a^1^ First shoulder; b^1^ second shoulder; a^2^ first shoulder; b^2^ second shoulder; c^2^ Veit–Smellie–Mauriceau. **C** Individual values regarding the maximal applied force in complicated deliveries with shoulder dystocia. A large scattering is shown using the different maneuvers within a scenario of shoulder dystocia in all-fours positions and using the Bickenbach and Veit–Smellie–Mauriceau maneuver in lithotomy position. The performance of each maneuver was operator dependent [Kruskal–Wallis test *p* < 0.01]. [N] Newton

Another experimental setup focused on the complicated deliveries and tested maneuvers in case of a shoulder dystocia in all-fours (Fig. 4A) and lithotomy position (Fig. 4B). Shoulder dystocia in breech delivery is a situation, where the trunk is delivered; however, the fetal arms are raised up and further descent can only be achieved through obstetrical maneuvers to free the arms. As described by Louwen et al. [12], in all-fours position the shoulder dystocia was solved using two rotational maneuvers followed by the Frank’s Nudge maneuver to deliver the head. In lithotomy position, we used the Bickenbach maneuver to resolve the shoulder dystocia (as described in Supplement 1) followed by the Veit–Smellie–Mauriceau maneuver for the delivery of the head. The comparison between both is shown in Fig. 4C.

The highest impact on the fetal neck region was recorded for the Frank’s Nudge maneuver in all-fours position (mean force 116.78 N, standard deviation 15.57 N) (Fig. 4A, C) and the delivery of the first/posterior arm in lithotomy position (mean force 127.98 N, standard deviation 22.27 N, *p* = 0.01) (Fig. 4B, C).

## Discussion

We used a simulation model to measure forces on the fetal neck during breech delivery. This is to the best of our knowledge the first analysis on the impact of birthing positions and maneuvers during breech delivery on the fetal neck. We could show that the applied force was lowest in uncomplicated deliveries in all-fours position. As soon as interventions were necessary, the force acting on the neck increased, as well as with the fetal weight and the number of maneuvers applied.

Several studies have indicated that vaginal breech delivery is one of the main risk factors for brachial plexus palsy due to complicated head delivery and traction on the fetal neck. The meta-analysis by Van der Looven et al. [11] was performed to identify risk factors for permanent brachial plexus injury. Spontaneous breech delivery was a significant risk factor (OR 2.49; 95% CI 1.67–3.7; *I*^2^ = 70%). Lalka et al. [10] described breech delivery as one of the strongest predictors for brachial plexus palsy (OR 15.38; 95% CI 5.60, 42.25). These studies show that brachial plexus palsy is an issue in breech delivery and should become an integral part of future training for spontaneous breech delivery, potentially directly measuring forces applied in a simulation model.

The retrospective cohort study by Louwen et al. [12] investigated real-life deliveries with breech presentation in an upright versus lithotomy positions and compared them to elective cesarean section. They found fewer neonatal birth injuries and a decreased secondary cesarean section rate for delivering breech in an upright position versus lithotomy. The limitation of the study was the small sample size of the control group: 229 deliveries in the upright and 40 in the lithotomy presentation [12].

Most studies of breech deliveries are retrospective and based on registry data. This makes comparisons between the different delivery methods difficult.

We used for the first time a full body high fidelity obstetrical simulator (SimMom, Laerdal), to investigate the force applied on the fetal neck during delivery in fetal breech presentation. We did not find comparable studies evaluating the forces affecting the fetus during vaginal breech delivery. There are only studies analysing forces on the fetal head or the brachial plexus during cephalic presentation. Allen, Sorab and Gonik [15] indirectly measured the forces, which were applied in real-life vaginal deliveries (cephalic presentation) using force-sensing devices attached to the provider’s delivery hand. They found that forces were typically about 47 N for routine deliveries, 69 N for difficult deliveries and 100 N for shoulder dystocia. A single case was reported where a force of 99.89 N resulted in a transient brachial plexus injury [16]. Another study was designed by Allen et al. [17]. They developed a birthing model and a microcomputer data acquisition system. This was used to measure applied forces during vaginal cephalic delivery for routine, difficult and shoulder dystocia cases. The physicians obtained 84 N for routine deliveries, 122 N for difficult and 163 N for shoulder dystocia deliveries (*p* < 0.002). Force levels exceeding 100 N were reached for many physicians during emergency situations [17]. The authors designed another trial and used a force-measuring birth simulator, which consists of a supported overhung beam and was equipped with sensors. Clinicians simulated again routine, difficult and shoulder dystocia deliveries [18]. Average peak forces of 68 N for routine deliveries, 118 N for difficult and 172 N for shoulder dystocia were revealed [18].

The absolute numbers of the above-mentioned three studies cannot be directly compared with ours, because they used indirect methods, whereas in our study the forces were directly measured within the fetal neck. Another difference of their study and ours is the fetal position (cephalic vs. breech presentation). However, measurements above 100 N should ideally be avoided as such high values were associated with emergency situations.

The lowest force in this simulation study could be recorded for the spontaneous delivery in all-fours position without the need of any further maneuver. We could demonstrate that using any of the described maneuvers, a higher impact of applied force was recorded. This can be explained by a higher tension on the fetal body during the maneuver, whereas in all-fours position within an uncomplicated delivery only gravity influences the applied force. As shown in Fig. 3B, the affecting force in all-fours position clearly depends on the fetal weight. If optimally performed, maneuvers in lithotomy position led to forces similar to those detected in uncomplicated all-fours position. However, as shown in Fig. 3B, the performance of each maneuver depends on the conducting physician and shows a significantly higher variation regarding each individual performance and level of training compared to uncomplicated all-fours position. Therefore, maneuvers during breech delivery should only be applied if absolutely necessary and need adequate training.

As soon as the fetal head had to be delivered without contractions, the applied forces on the fetal neck increased. In conclusion, as long as there is no emergency situation, e.g., fetal bradycardia, a delivery with support of contractions should always be preferred to avoid unnecessary higher acting forces.

It is interesting that, within the complicated deliveries, the Veit–Smellie–Mauriceau maneuver is associated with the lowest force on the fetal neck region. This can be explained by the fact that the physician directly grasps the fetal head and thereby prevents any traction on the fetal neck. On the other hand, potential soft tissue injuries of the oral region have to be considered, especially when an index finger is introduced into the mouth rather than two fingers placed on the cheek bones.

It was interesting to note that we detected the highest forces applied to the fetal neck region during cases of shoulder dystocia, for both all-fours and lithotomy position. However, the time point where the maximum force occurred was different for these two positions (Fig. 4C).

In the lithotomy position, we detected the highest forces while resolving the shoulder dystocia (mean value 127.98 N, SD 22.27 N). Interestingly, in all-fours position, the maximum force was applied during the delivery of the head (mean value 116.78 N, SD 15.57 N). This is important information and should help us to specifically focus training on the most dangerous situations.

A strength of our study is the use of a high-fidelity obstetrical simulator and Birthing Baby including internal built-in sensors which made direct recordings possible. A further strength is the use of Automatic Delivery Module 2 capable of standardizing contraction forces. This simulation is capable to reflect almost any emergency situation. However, this model only provides the opportunity to measure forces applied to the fetal neck. We decided to use the SimMom simulator and Birthing Baby together with PROMPT Flex-Software, instead of the PROMPT hemi-pelvis. The advantage of the SimMom is the flexibility to simulate different birthing positions and to be able to put the simulator in all-fours. Additionally, this standardized model does not reflect individual patient characteristics such as anatomical differences, pelvic floor function and fetal size.

Our proposed simulation model and our systematic analyses can be used to better train and reproduce individual situations and systematically evaluate the impact of obstetrical maneuvers. Future research should concentrate on the demonstration of how the forces applied to the fetus change with training and how feedback can improve the individual performance, reduce damage and optimize conditions for safe vaginal delivery.

## Conclusion

The novel proposed simulation model has enabled us for the first time to directly measure forces acting on the fetal neck. This made recordings of forces in the two birthing positions and the various maneuvers of the breech delivery possible.

In uncomplicated breech deliveries, the use of the all-fours position is associated with a lower level of applied forces on the fetal neck compared to delivery in lithotomy.

## Supplementary Information

Below is the link to the electronic supplementary material.

Supplementary file1 (DOCX 15 KB)
