# Supplementary material for: Comparing forces on the fetal neck in breech delivery in lithotomy versus all-fours position: a simulation model
Source: Arch Gynecol Obstet. 2022 Jul 20;308(1):91–9. doi: 10.1007/s00404-022-06671-5 (PMC10191913; doi:10.1007/s00404-022-06671-5)
Supplement: Supplementary file 1 — Supplementary file1 (DOCX 15 KB) [file 404_2022_6671_MOESM1_ESM.docx]

Supplement 1

**Obstetrical Maneuvers**

All-fours – spontaneous delivery:

Uncomplicated delivery in all-fours position is performed without application of external force or maneuvers.

All-fours – Frank´s Nudge:

Gentle pressure is applied on the anterior part of both shoulders in order to deliver the head as described by Louwen et al.^1^.

All-fours – Rotation maneuver for shoulder dystocia:

As described by Louwen et al. ^1^ in all-fours position the shoulder dystocia is solved by using two rotational maneuvers (180° and 90° backwards, the face of the baby always directed towards the operator and the back posteriorly) followed by the Frank Nudge maneuver

Supine – Normal delivery (Bracht maneuver):

The fetal trunk and legs are grasped with both hands and are levered up and around the mother’s symphysis in order to deliver the fetal head slowly.

Supine – shoulder dystocia (Bickenbach maneuver):

The legs are raised and the posterior arm is gently freed by wiping the arm out coming from the back, the legs are then lowered and the upper arm is gently freed by wiping it out again coming from the back. After delivery of the fetal body and shoulders, the head was delivered with the Veit Smellie Mauriceau-maneuver: the obstetrician places the index and middle finger on the fetal cheekbones and the other hand on the fetal head near the occiput (keeping the head between the two hands) while the baby is placed on the obstetricians forearm. The head can now be moved slowly downwards until the occiput becomes visible. Then the trunk is elevated and levered over the mother’s symphysis.

REFERENCE

1. Louwen F, Daviss BA, Johnson KC, Reitter A. Does breech delivery in an upright position instead of on the back improve outcomes and avoid cesareans? *Int J Gynecol Obstet*. 2017. doi:10.1002/ijgo.12033
